# Novel Method for Failure Modes Detection in UV-Cured Clear Coated Polymer for Automotive Interior Mechatronic Devices

**DOI:** 10.3390/polym14183811

**Published:** 2022-09-12

**Authors:** Ion Cristian Braga, Razvan Udroiu, Anisor Nedelcu

**Affiliations:** Department of Manufacturing Engineering, Transilvania University of Brasov, 29 Eroilor Boulevard, 500036 Brasov, Romania

**Keywords:** failure modes and effects analysis (FMEA), polymers, coating, double shot injection molding, design of experiment, defects, multi-step process

## Abstract

Plastic parts used in automotive interior are difficult to coat, due to their low surface energies as well as their sensitivity to temperature and solvents, rendering the development of coating systems for such substrates challenging. Automotive customer requirements are explicit and clear, mainly focused on functional and surface defects. A new failure modes detection methodology of UV clear coated polymers for automotive interior, obtained by a multi-step manufacturing process, is proposed. The polymer complex parts analyzed in this paper are manufactured in various steps as follows: two components plastic injection molding, primer coating, laser engraving, and UV-cured clear coating. The failure modes detection methodology of the parts within each process step is investigated using different tests and analyses as follows: surface tension test, painting adhesion test, optical 3D measuring, energy dispersive X-ray analysis (EDX), and microscopy. A design of the experiments (DoE) based on the Taguchi technique with the aim to detect the influence of the main factors that lead to surface defects was performed. The proposed methodology is validated by a case study. The results showed that the mold temperature and the laser engraving current have a significant influence on the surface defect occurrence. Additionally, a possible contamination of the molding tool can generate the defects. A solution to reduce the occurrence of the failures was proposed, reducing the defect rate from 50% to 0.9%.

## 1. Introduction

Currently, the interior of cars is becoming more and more sophisticated and customer requirements are increasingly referring to the colors and surfaces of the elements that are in the passenger compartment [1]. Thus, the requirement for surfaces to be glossy and velvety comes as a challenge for car assembly lines. In addition, the reliability of car components increases from year to year and the guarantee offered to users is an advantage in sales. Some of the properties of thermoplastic polymers used in the automotive interior are significant: noise and vibration damping, weight reduction, and recycling. Thus, the polymers are increasingly used in the automotive market to reduce pollution by reducing car weight (replacing metals) and ultimately reducing fuel consumption.

The clear coating technologies used in the automotive field allow the increases the resistance of surfaces to scratching, abrasion, discoloration, or contamination [2]. These coatings are based on thermosetting resin-based raw materials that can be cure under UV or heat exposure. The most common polymer substrates that can be coated in automotive industry are the thermoplastic polymers such as ABS, PC, or ABS/PC blends. The materials’ properties that are relevant for the coatings technologies and that can produce effects on properties of the coated plastic part are mechanical behavior, thermal behavior, solubility, electrical behavior, and surface characteristics [1].

A major number of complaints associated with symbol marking, scratch, and paint imperfections on the surface must be avoided by performing good clear coating parts that are mandatory for Original Equipment Manufacturers (OEMs) [3]. Various defects can occur at each stage of the process, leading to rejection of parts. Automotive customer requirements are explicit and clear. They are classified into two categories: functional defects (e.g., button lock, cumbersome assembly, etc.) and surface defects or appearance elements (e.g., scratched part, paint defects, etc.). There are, additionally, some surface defects or so-called subjective surfaces such as sink marks, weld lines, pin holes, fish-eye, orange peel texture, scratches, wrong glossy level, and wrong shade of color. The causes of the defects of polymeric parts obtained by a multi-step manufacturing process, such as plastics injection processes and their subsequent processes, e.g., painting with transparent varnish hardened, are crucial to be investigated.

Cuevas et al. [4] characterized the polyurethane clear coats applied by a conventional spray coating method on PC/ABS substrates, but the surface defects were not analyzed. Rungwasantisuk and Raibhu presented the variables in the UV curable coating on polymer substrate [5] and how to reduce the defects, but mainly the contamination-caused ones. A deflectometry based defect detection system used for large painted automotive parts was proposed in [6], used mainly to detect the various manufacturing defects induced during the molding and painting processes. Vieira et al. [7] proposed environmentally friendly plastic over-injection equipment for the automotive industry to cover the terminations made of Zinc alloys (so called Zamak) of the flexible cable, named Bowden cable, with plastic. The defect analysis was not performed.

The possible surface defects can occur in all the manufacturing steps of coated plastic parts. Thus, all the manufacturing steps, including plastic injection molding, primer coating, laser engraving, and coating, have to be analyzed. A lot of research papers are focused on the analysis and optimization of the injection molding parameters and how these affect the properties of the injected parts [8,9,10], but a failure modes and effects analysis was not included. Thus, in [9,11] the injection parameters that can affect the characteristics of surface quality and strength, such as the injection speed, mold temperature, melt temperature, injection and holding pressures, and cooling setup and time, were analyzed. Moayyedian et al. [12] used Artificial Neural Network and Taguchi Techniques, aimed to find the optimal parameters for injection-molding process for polypropylene parts, with minimum defect possibility. They conclude that the end-product quality was mostly influenced by filling time, followed by the pressure-holding time. Additionally, the effect of moisture in plasticization parameters has been taken into consideration by Chen et al. [13], but not the surface defects that occurred.

Moon et al. [14] focused more on the damage mechanism that led to scratches in the lifetime of the products and UV degradation due to exposure to sun, but not to the defects that occurred in time of the coating. About the contamination defects, Biçen and Güvenç focused on the crater formation on coatings and their causes, but not to fish-eye defects [15]. The UV curable powder coatings were studied by Czachor-Jadacka et al., and the result shows low probability of the surface defects, but the possibility of their usage on the low thermal surface such us polymers is to be investigated [16].

Failure modes and effects analysis (FMEA) [17,18,19,20,21] is a risk management tool that identifies and assesses the causes and effects of potential failures in a system or processes before they occur. Failure mode, effects, and criticality analysis (FMECA) is an extended version of FMEA that allows decision-makers to allocate resources adequately by using the risk priority number to prioritize the failure modes [18]. A failure mode and effect analysis performed in [22], focused on the performance of an injection molding machine, showed that the implementation of the methods increases the Overall Equipment Effectiveness rate and has a positive impact on the company. The risks that could occur during the plastic injection process with traditional FMEA and Grey Relational Analysis (GRA) was presented in [17]. Dimensional defects were identified as a highest risk.

From the literature survey results, it is shown that the FMEA method was applied in injection molding manufacturing process, but the studies are not focused on complex multi-step manufacturing process such as injection molding, engraving, surface coating, etc. The implementation of a FMEA methodology in production of polymer complex parts for automotive industry, having benefits in terms of cost reduction and shortening of the time-to-market in products, plays a key role. Thus, the detection of defects, finding the root causes, and eradicating them leads to the scrap rate reduction of plastic parts.

The paper aims to propose a new methodology for failure modes and effects analysis of polymer parts obtained in multi-step manufacturing process and to analyze the defects in plastics injection processes and, subsequently, in their painting with transparent varnish hardened by UV treatment, focused on surface defects or so-called subjective surfaces defects such as pin holes and fish-eye.

## 2. Materials and Methods

### 2.1. New Methodology for Failure Modes Detection of Polymer Parts

The main objectives of the new methodology in Failure Modes detection of polymer parts are to define quality tools based on standards for assessment complex parts for the automotive industry. This methodology includes experiments, statistical analysis, results interpretation, and the identifying of possible changes in the processes. The automotive interior mechatronic devices are produced in a flow with various process steps from plastic injection molding, coating, electronic production surface mount technology and through hole technology, laser engraving, assembly, and final test. This paper is analyzing the causes of the possible defects that can occur in UV clear coating of injection plastic parts, which are dependent on previous steps of the manufacturing process. The process steps that can lead to those defects are as follows: two components plastics injection, colored primer coating, laser engraving, and UV-cured clear coating.

**Figure 1:**
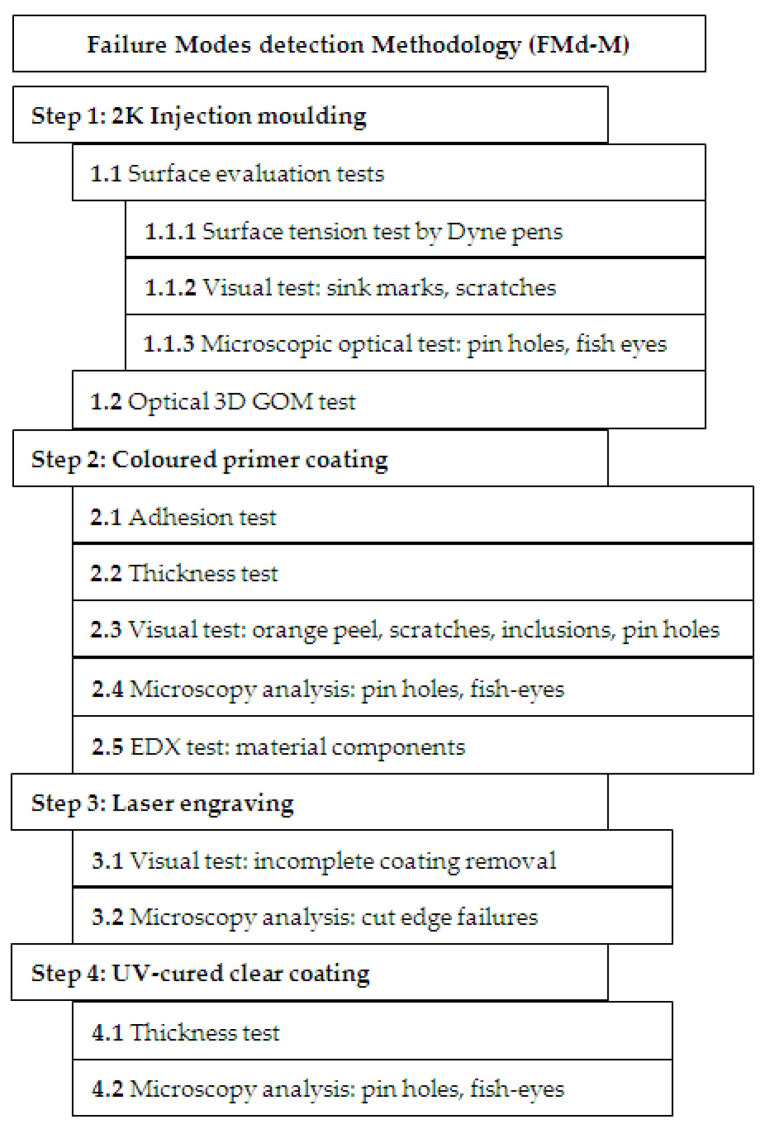
Novel Failure Modes detection methodology.

The current testing methods of defects on coatings are based on only one stage of the process [15]. In addition, surface evaluation quality of injection molding requires surface tension assessment and visual testing with the naked eye or a magnifying glass. In the coating process steps, only adhesion test, thickness test, and visual test are currently used. The advantage of the new method is the use of the microscopic analysis within all stages of the process as well as the 3D optical scanning test. The main advantage of the proposed method is to consider the process steps as a whole process, with the risks associated with factors that originate in the initial steps and evolve during the other steps. To improve the detection of defects, for a better understanding of the failure method that can lead to finding the root causes and eradicating them, and to reduce the scrap rate, a new failure modes detection methodology of the complex polymer parts is proposed (Figure 1).

Two phases were taken into consideration for a complete defect analysis method of plastic injection molding to UV-clear coated parts. A defect mode detection methodology based on the process steps was proposed in the first phase. In the second phase based on the output data from the methodology and statistical methods, the root causes leading to these failure modes and the prevention actions for the occurrence were found. The equipment used for the various tests to ensure the detection of the failure modes are the following, and their roles are described below: Dyne Pens 20-PENQAPLUS, Optical Microscope type ZEISS O-INSPECT 5/4/3, ATOS Scan Box—Optical 3D Coordinate Measuring Machine, Series 4, ATOS CORE MV 135, Ultrasonic thickness meter TMT-TM-1240, Cross Hatch Cutter 1 mm—X2001, and Electron microscope with EDX—HITACHI S2600N.

In the first stage of the proposed methodology, tests about injected molded parts were performed, such as a surface evaluation test, and then optical measurements. The surface evaluation test consists of surface tension test, visual test, and microscopic analysis. The surface tension test was performed using Dyne Pens 20-PENQAPLUS (Dyne Testing Ltd., Lichfield, UK) [23]. For the visual test, in the case of the material being white, the plastic parts are painted with a black spray and checked under a magnifying glass, which increases the probability of detecting defects and, implicitly, reduces the risk in FMEA regarding detection of this one.

The injected parts have been analyzed by Optical Microscope Type Zeiss O-Inspect 5/4/3 (Carl Zeiss Industrielle Messtechnik GmbH, Oberkochen, Germany) [24], to understand if there some factors that can lead to next step in fish-eye defects occurrence can be seen in the primer coating. The optical measurements of the parts were performed using an ATOS Scan Box Optical 3D Coordinate Measuring Machine, as type ATOS CORE MV 135 (GOM Metrology, Braunschweig, Germany) [25].

**Figure 2:**
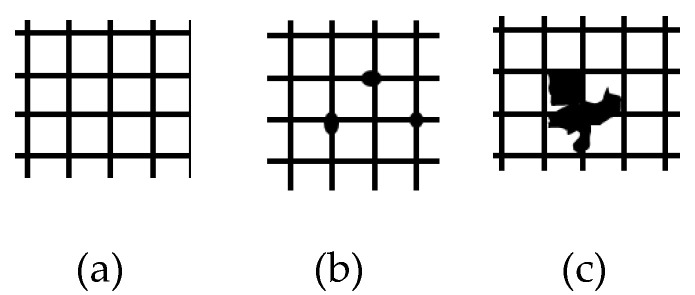
Evaluation levels representation of the adhesion test, adapted based on ref [26]: (**a**) low level (level 0); (**b**) maximum accepted level (level 1); (**c**) high level (level 4).

Five different tests and analyses are performed in the second stage of the methodology, after the parts are colored primer coated (Figure 2), as follows: adhesion test, thickness test, microscopy, and energy-dispersive X-ray (EDX) analysis.

The adhesion test was performed using the device Cross Hatch Cutter 1 mm—X2001 and based on the standard DIN EN ISO 2409:2020, Paints and varnishes—Cross-cut test [26], having six levels of evaluation. Based on the mentioned ISO standard and customer-specific requirements, the certain level is the one used as reference, for the study case presented that the level 1 was the maximum accepted for the adhesion test. It means the traces on the adhesive tape used for this test must be less than indicated in the Figure 2b, level 1.

The thickness of the primer was measured on metallic plates used in the same painting spraying system with the same parameters as the one for the coated plastic parts, by measurement device ultrasonic thickness meter TMT-TM-1240 (OCS.tec GmbH & Co. KG, Neuching, Germany), performing a set of 3 measurements on close areas and registering the average of the measurements. A Gage R&R study was performed [27] taken into consideration all three inspectors who make the measurements [28].A preliminary microscopic investigation of the possible surface defects was performed using an optical microscope. In this research, a Zeiss O-Inspect 5/4/3 microscope was used. A deep analysis of the sample injected molding parts coated with the colored primer need to be performed in order to analyze the microstructure and determine information about the chemical composition of the samples in the defect areas. Electron microscope SEM-EDX Hitachi S2600N (Hitachi, Tokyo, Japan) was used for this analysis.

In the third step of the proposed methodology, the laser-engraved parts are visually analyzed for detection of incomplete coating removal, and then microscopy investigations were performed.

After the last processing step (fourth step of the methodology) of the samples, consisting of UV-cured clear coating, a thickness test and microscopy analysis were performed.

A design of experiments (DoE) with the purpose to detect the influence of the main factors that lead to surface defects and to segregate them was performed. In order to perform the design of experiments, the Taguchi method was selected, taking into consideration the big number of the factors supposed to influence the final result and to be linked with the fish-eye defect occurrence. Therefore, the influence factors and their levels were selected (Table 1).

The following 8 factors were chosen: injection molding temperature, injection molding pressure, 3D shape, surface energy of the parts to ensure the right coating, primer thickness (opaque coat), the laser engraving power, the clear coat UV-cured thickness, and the power of UV lamps used for curing. These factors were selected because of their possible influence on surface defects. Injection molding temperature and pressure are the most important factors with effects on plasticization and cooling, ultimately, as well as 3D shape and surface energy, all of which are related to possible surface defects. The thickness of the primer or clear coat could show the influence of the coating in the appearance or development of surface defects. Laser etching current and UV lamp power could have an influence by increasing the local temperature, considered for many surface defects. For each factor, three levels were selected: low, medium, and high, with the values presented in Table 1. The levels values of each factor were selected based on the recommendations of the material data sheet (temperature and pressure in the mold), of the laser engraving supplier (laser current), and the coating system supplier (thicknesses and UV power). The 3D shape and surface tension values were chosen based on the worst, average, and best parts from a production run with changes in material batches, mold cavities and different operators handling the injection molding machines.

Using Minitab 19 software (Minitab, Ltd., Coventry, UK) [29], the data were analyzed, and the Signal to Noise Ratios (S/N) were calculated, which is a measure of robustness. Finally, based on the DoE and analyzing with the help of the cause–effect diagram, the factors that can lead to the occurrence of this defect are discussed.

### 2.2. Materials and Process Specifications

In this work, a 2K injection molded component from a mechatronic device, used in automotive interior, has been selected as the benchmark. The part consists of a polycarbonate/acrylonitrile butadiene styrene (PC-ABS) blend type Bayblend T65 XF of black color, as base material, over which is injected a PC material type Makrolon 2407, translucent, with UV stability properties by using the absorbers to dissipate the light energy from UV rays. The polymers were injected in two phases as follows: in the first phase, the support material, then the translucent material. 2K injection molding, known as double injection, is an innovative manufacturing process used to produce complex molded parts from two different materials into the multi-chambered mold. An injection molding machine type Sumitomo Systec 350–320 h/200 v (Sumitomo (SHI) Demag Plastics Machinery GmbH, Schwaig bei Nürnberg, Germany), hybrid toggle, with two injection units, horizontal and vertical, was used. The injected molding plastic 2K parts are produced in a tool with 4 + 4 cavities in 2 phases. In the first phase, the black base material is injected into 4 cavities (numbered 1 to 4), then the mold is rotated 180 degrees, and in the second phase the translucent white material is injected over the black. After rotation, the black material is injected into the next 4 cavities (numbered 5 to 8), then rotated 180 degrees and so on.

Makrolon 2407 plastic was coated with a primer, engraved with a laser system for the symbols indicating the operation of the mechatronic device buttons, and finally covered with colorless UV-cured paint.

The painting process is by two layers sprayed in an automated installation type Venjakob (Venjakob Maschinenbau GmbH & Co KG, Rheda-Wiedenbrück, Germany). The first layer is a colored primer type Alexit (Mankiewicz, Hamburg, Germany) afterwards, the drying takes place in an oven heated to 80 degrees Celsius for 40 min. Subsequently, the parts were laser engraved for symbols using a machine type Powerline E20 (Rofin-Sinar Laser GmbH, Hamburg, Germany), using a YAG laser pulsed with 1064 nm wavelength. Then, the parts were coated again in the Venjakob installation, with a layer of clear varnish type Alexit, with dual-cure technology, IR drying (infrared), followed by a curing with UV polymerization, and drying in the oven at 80 degrees Celsius for 40 min, as the coating system supplier recommended for best results and to prevent solvent residue that could lead to color changes in car use over time.

## 3. Results and Discussions

### 3.1. Results of the Proposed Methodology Applied on the Automotive Polymeric Component

**Figure 3:**
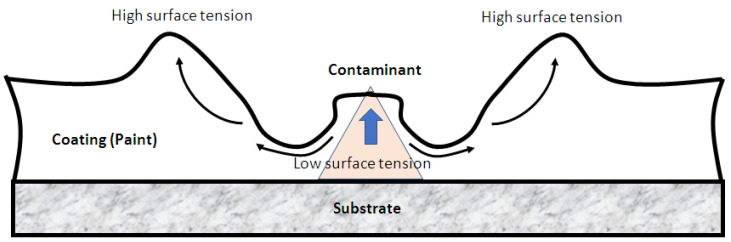
Contamination defect called as fish-eye defect.

Based on the proposed methodology, one very rarely occurring failure mode was detected as very important and at a high level of scrap, the so-called fish-eyes defect. This defect is caused by a contaminant with low surface energy, from which the applied coating is displaced, as shown in Figure 3 [30,31].

Based on the FMd-M methodology, the mentioned tests were performed and the result was collected (Table 2).

Samples were taken at the level of eight paint frames, containing two sets of left-right paired frames, two frames taken from the left end of the painting line, two frames from the middle-left, two frames from the middle-right, and two frames from the right end of the painting line. This sampling mode was selected in order to follow—based on the detection of defects on the respective frames—whether the parametric spraying factor is an influencing factor in the occurrence of this type of defect.

**Figure 4:**
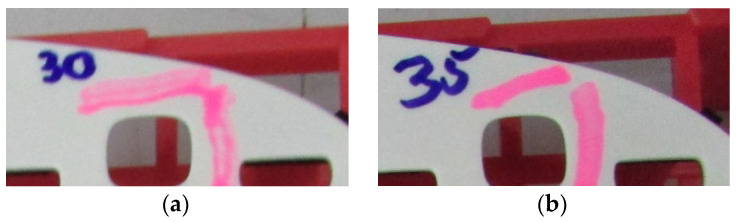
Surface tension value is (**a**) 30 mN/m for bad parts; (**b**) 35 mN/m for good parts.

The surface of the parts resulted from the injection molding process were analyzed by Dyne Pens, and the results about the surface tension test are shown in Figure 4. The affected areas of the parts are determined for a 30 mN/m surface tension related with the coated parts which present the fish-eye defect. The parts coated without the fish-eye defect have been the one measured at 35 mN/m surface tension.

To improve the adhesion of the surface by using corona discharge or low-pressure plasma treatments [32] cannot be an affordable industrial solution in case of injected plastic parts due to expensive processes designed to handle the parts between process steps. A possible solution to increase the adhesion by surface preparation could be the cleaning with a solvent [33] or even cleaning with dry ice CO_2_ blasting [34].

**Figure 5:**
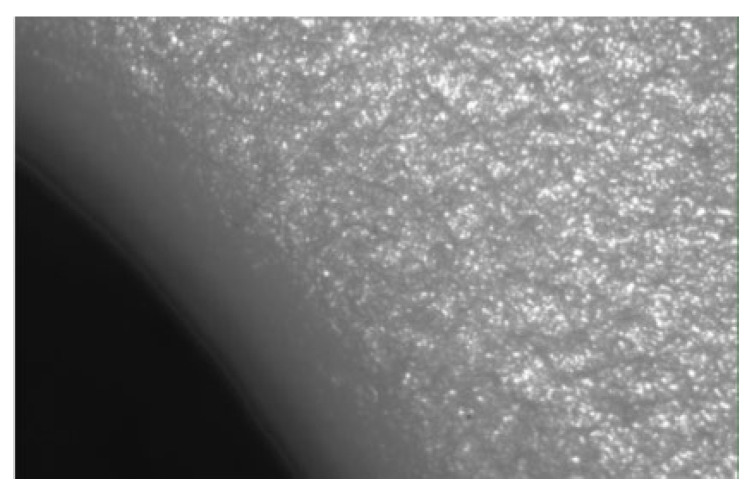
Microscopic analysis of injected parts.

There were no defects detected from the visual and microscopic analyses (Figure 5).

**Figure 6:**
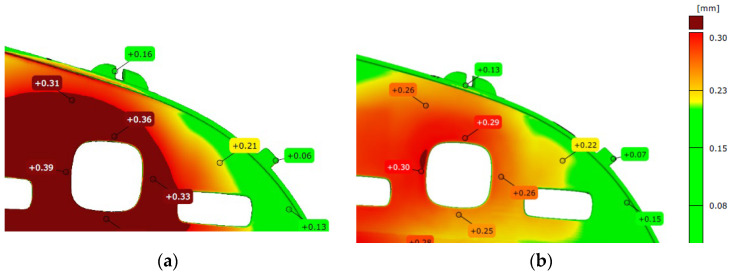
The results of optical measurements of: (**a**) a part out from the cavities 3, 4, 5, and 6; (**b**) a part out from the cavities the cavities 1, 2, 7, and 8.

The parts produced in the eight different cavities of the mold were optical 3D measured to investigate their 3D shape. This factor was taken into analysis, supposing that the surface energy will be influenced by the 3D shape. A sample of three parts has been measured by optical ATOS Scan Box machine. The measurement results show that the parts out from the cavities 3, 4, 5, and 6, from the eight cavities of the mold, were detected with bigger deformations compared with the ones from the cavities 1,2, 7, and 8 (Figure 6). These results do not lead to a clear link with the possible failure mode of fish-eye defects occurrence.

**Figure 7:**
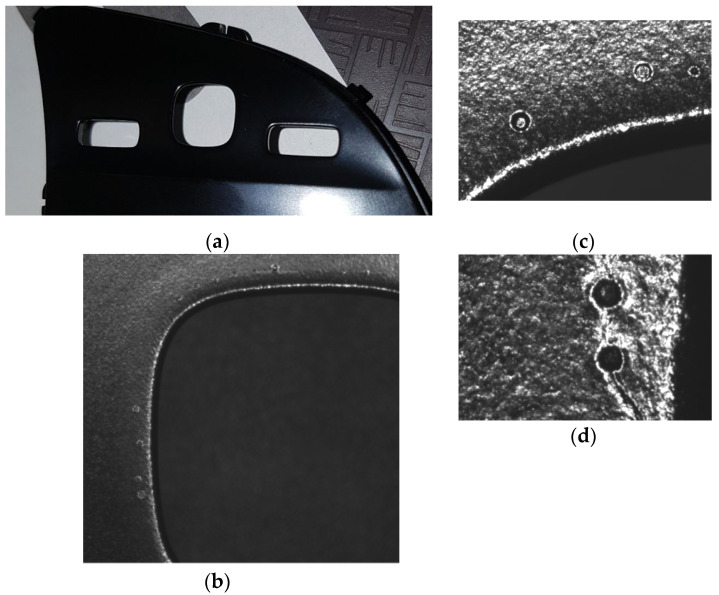
Coated parts analysis: (**a**) Visual test; (**b**) Optical microscopy analysis of the rectangle window of the part; (**c**) Optical microscopy analysis on the upper zone of the rectangle window; (**d**) Optical microscopy analysis on the right zone of the rectangle window.

The coated parts with the black primer are visually inspected under the magnifying glass and under light conditions according to the requirements of the VDA16 standard [35] to detect defects such as intrusions (due to contamination during spray or drying stages), needle holes, incomplete paint, too much paint on the corners, orange peel, and scratches (Figure 7a). The parts that have the defects mentioned above were segregated (according to the normal production process, a percentage between 0.3% and 1.5% is normal to be detected, and impurities involved during spray cannot be prevented).

The optical microscopy investigations of the coated parts, used to understand the mechanism of the failure, have shown the occurrence of the defects on the plastic parts first layer coating, on the nearest area of the rectangle windows from the parts. Investigation led to fish-eye defect on the primer painted parts (Figure 7b–d).

The results of adhesion test have shown a good bonding over all parts selected in the experiments, the level of evaluation being zero—Level 1 with very good bonding, and no traces from the cut paint on the tape.

The result of the thickness test shows that it is not related to the “fish-eye defects” on the plastic parts because the paint thickness was measured to be in the tolerance range, close to the average value of 17 microns.

**Figure 8:**
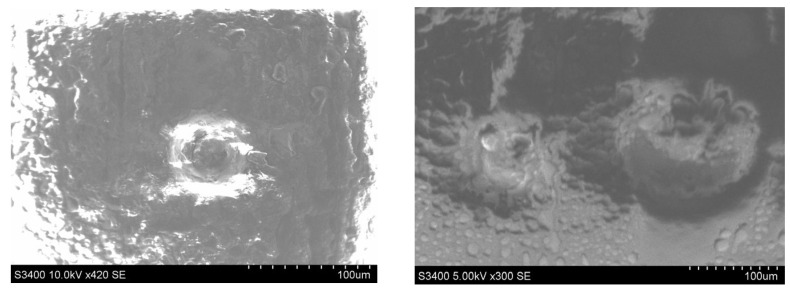

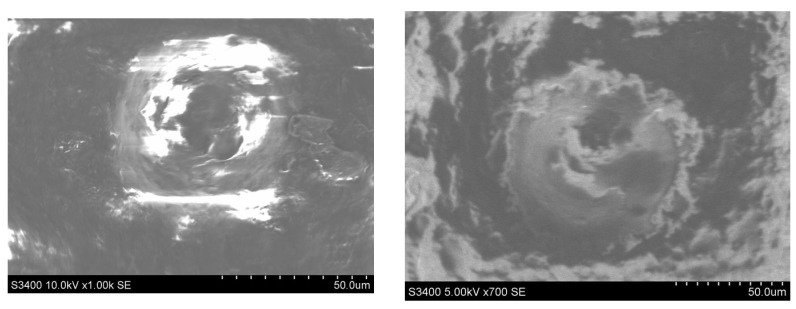
SEM microscopy analysis study in different four areas of the part.

A SEM microscopy analysis study was performed in different four areas to deeply analyze the fish-eye defects on the nearest area of the rectangle windows of the part, as is shown in Figure 8. This defect is characterized by circular voids or crater-like openings. The size of the circular shape defect was in the interval 50 to 178 microns.

**Figure 9:**
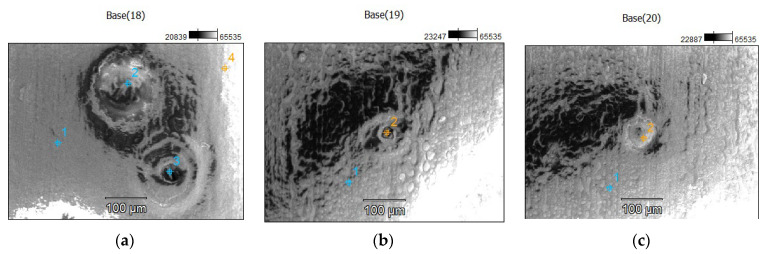
Choosing the analyzed EDX points (1, 2, 3 or 4) in three different zone with defects of the part: (**a**) EDX points in Base(18) area; (**b**) EDX points in Base(19) area; (**c**) EDX points in Base(20) area.

**Figure 10:**
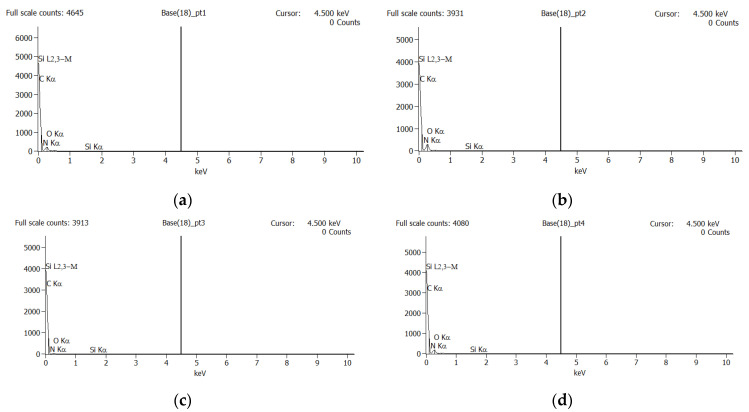
EDX analysis for the four points within the case (**a**) from Figure 9: (**a**) Point 1; (**b**) Point 2; (**c**) Point 3; **(d**) Point 4.

A number of three EDX analyses were performed within different areas which contain defects of the part, as shown in Figure 9. Four points were chosen for the first EDX analysis on the SEM image (Figure 9). Based on the results shown in Figure 10, only two EDX points are relevant (Point 1 and 2) for the following analysis. The results of the EDX analysis for the first area are presented in the Table 3. Similar EDX results, within only two points, were obtained for the other two interested areas from Figure 9. A possible contamination with a material such as oil or demolding spray during the injection process of the plastic part was checked by EDX analysis, and the results are shown in Figure 10.

The EDX analysis did not reveal any clear element that should not be present, because in the plastic substrate mainly hydrocarbons are present [16], and the base layer is also a hydrocarbon, so that the presumption of oil or release agent contamination was maintained.

**Figure 11:**
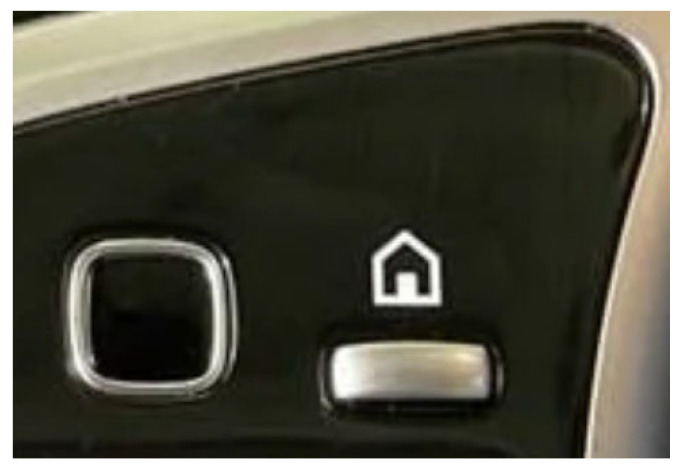
Laser engraving of the parts.

Laser-engraved parts after the black primer coating process are visually inspected to check that the paint has been completely removed. Microscopic analysis and measurements are performed to inspect the symbol line sizes as well as possible laser-etched edge defects (Figure 11). There were no defects detected from visual and the microscopic analysis, the process being optimized [36].

**Figure 12:**
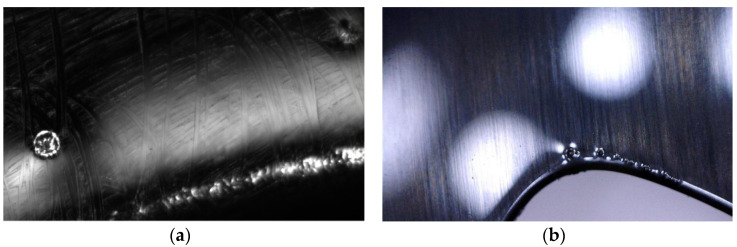
The issue (fish-eye) detected on the final clear coated parts: (**a**) in the area of the square windows; (**b**) at a distance from their edge.

The thickness of the UV-cured clear coating has been measured, similar to the coating with primer. The result shows that they are not related to the “fish-eye” defects that appeared on the plastic pieces, as in the case of the primer, the thickness is in the specified tolerance with an average of 27 microns [5].The “fish-eye” defects were detected on the UV-cured clear coating parts by the optical microscope, both in the area of square windows, but also at a distance from their edge (Figure 12a,b).

### 3.2. Design of the Experiments Results

The main factors that lead to surface defects were determined based on the Taguchi method. The response table for the Signal to Noise Ratios are used to identify control factor settings that minimize the effect of noise on the response, based on the “smaller is better” statement. Delta is calculated by the difference between the maximum average response and the minimum average response of the signal-to-noise ratio for each factor. Finally, the ranking shows the influence in the occurrence of the analyzed defects for each factor.

**Figure 13:**
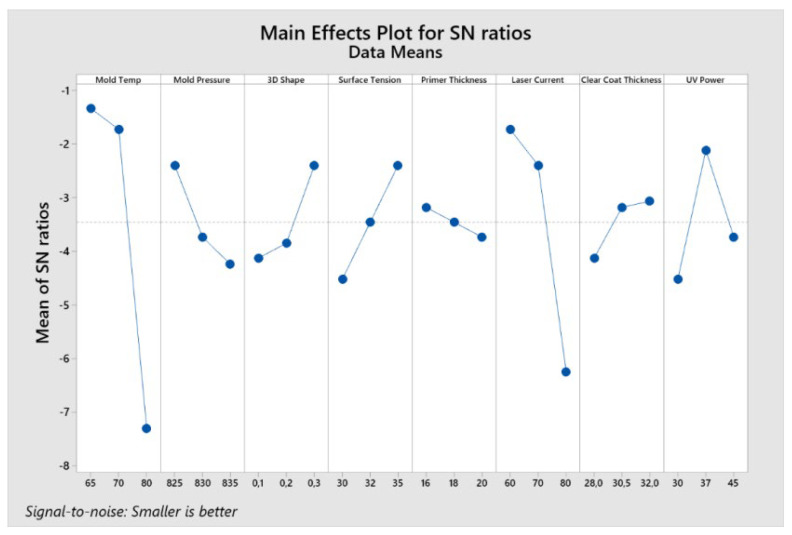
Main effects plot for Signal to Noise Ratios.

As can be seen, based on the ranking from the Table 4, but also in Figure 13, two factors have the most influence in fish-eyes defect occurrence: the mold temperature and the laser engraving current. In third and fourth places, the UV power and the surface tension could be selected as medium influence.

Therefore, based on the selected parameters and their levels, the DoE shows the optimal parameters combination to achieve the minimum quantities of the “fish-eye” defects as following: Mold Temp level 1, Mold pressure level 1, 3D Shape level 3, Surface Tension level 3, Primer thickness level 1, Laser current level 1, Clear Coat thickness level 3, and UV Power level 2. It must be underlined that the surface defects will be reduced by using the optimal combination, but not eliminated at all.

Finally, based on the DoE and analyzing with the help of the cause–effect diagram the factors that can lead to the occurrence of this defect, a possible contamination resulting in the plastic injection process could explain why the surface tension of the surface is lower, as well as why the number of defects increases with increasing injection temperature and/or etching current, as well as increasing UV curing power.

### 3.3. Failure Modes and Mechanism Studies

Based on the DoE studies, two factors were retained in the root cause analysis because their influence was greater than the other factors: die temperature and laser etching current.

As FMd-M results, hydrocarbon contamination revealed by SEM/EDX was one of the possible factors, mainly in the rectangular window of the part. The initial investigation of the root cause considered the analysis of the injection mold by disassembling it and by performing deep inspections of each metal part in the indicated rectangular area to detect some traces or indicators related to possible contamination, knowing that the cooling system was based on oils, but nothing was detected. As a result, a new analysis was ordered by checking specific parts of the cooling system in the area where the fish-eye defect was most often detected.

**Figure 14:**
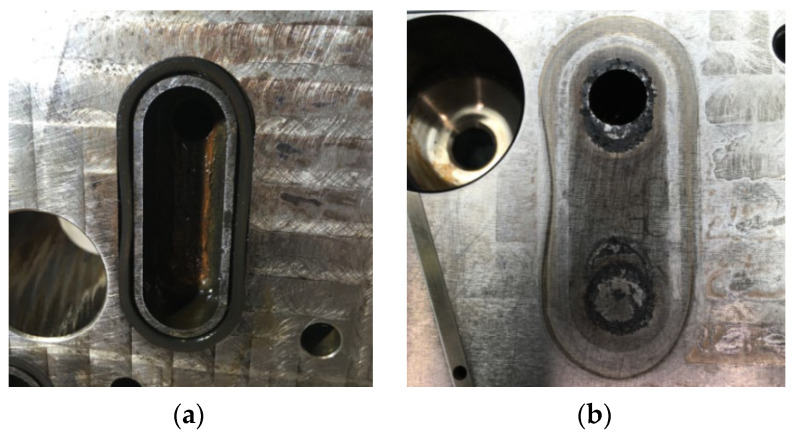
(**a**) Injection molding tool with the O-ring affected; (**b**) Traces generated by bad assembly of O-ring.

After re-analysis of both sides of the mold, checking the cooling system, some traces were detected in connection with a sealing “O” ring (Figure 14); the traces indicate that the escaped oil was drained at the location of the rectangular window and, due to the high temperature, was burned during the injection cycle and could not be detected as a clear contamination on the entire surface of the plastic part, not even by checking the surface tension.

**Figure 15:**
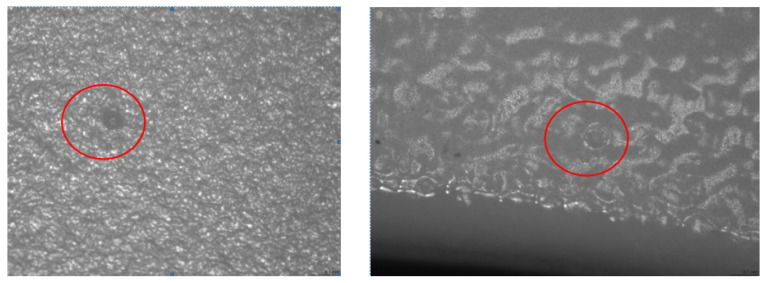
Optical microscopy inspection of the parts resulted using an increased mold temperature.

Finally, the root cause leading to this fish-eye defect was the oil contamination during the plastic injection cycle in the area near the rectangular window. Thus, the hydrocarbons penetrated into the structure of the amorphous material of the part. During the painting with UV-cured clear varnish, the hydrocarbons had the role of the contaminant factor with low surface energy that caused the occurrence of the defect. The hydrocarbons have evaporated during painting with UV-cured clear varnish, and the small bubbles have been blocked by the drying process of the material during polymerization. Therefore, increasing the mold temperature as a factor in DoE shows the influence in the evaluated parts. The third factor, the laser etching current, was also related to the local temperature increase during etching near the window area and released the hydrocarbons from the structure of the plastic material only on the surface, and then the bubbles began to move during the coating process transparent with UV polymerization. To prove the possible effects of oil contamination under pressure, the defective “O” ring was reassembled and batches were produced with an increase in the mold temperature, and the window area was investigated. As shown by optical microscopy inspection, small circles were detected on the white surface of the plastic, and this cannot come from a normal amorphous structure of the material (Figure 15).

Some solutions to reduce then to eliminate the occurrence of the failures were found as follows. A defect rate indicator was used to measure the results of all counter measures and improvements actions, based on the report between the number of parts that contain one or more fish-eye defects and total parts checked, in percentage. As a short-term improvement action, the tempering step was implemented at 80 to 85 degrees Celsius during 2 h of the injected parts before the primer painting process. It leads to reducing of the defect rate from 80% to 50%. Furthermore, the changing of the cooling system of the injection molding tool from oil to water led to reducing of the defect rate from 50% to 0.9%. Variation of the defect rate of 0.9% generates the need to investigate the plastic parts, and new parameters in injection molding reduced the scrap rate from 0.9% to 0.05%.

## 4. Conclusions

This paper investigated the surface defects in UV clear coated automotive polymer parts, based on a novel method for failure modes detection. The following conclusions can be drawn:

- The development of coating systems is a necessity based on the fact that many polymers are difficult to coat. There is a need for good surface quality for UV-coated plastic parts used in automotive interior mechatronic devices.
- The methodological checking after each process step is recommended in order to catch the known or not-known defects and to understand their evolutions and/or influences due to all parameter changes.
- Support of the specialists in development of the cause–effect factor relationship for each type of not-yet-known defects is also recommended.
- The surface defects such as fish-eye defects can occur within UV-cured clear coating process or due to mold contamination with either oil or silicone compounds. The mechanism of generation of this defect is based on the difference in surface tension between the contaminant and the coated surface, with molecules starting to migrate towards the surface of the coating.
- The root cause analysis started from the very beginning, from the injection molding tools. After deep analysis of both parts of the tool, some traces were detected in link with an O-ring that could have a possible effect; escape pressured burned oil in time of the molding cycle and contaminated the structure of the plastic part in that window area. As a result, in order to prevent this type of defect from occurring, action must be taken to prevent contamination. There are several methods to clean the substrate, such as: wiping with a solvent using a rag, or in the automatic coating line using carbon ice, ionized air.
- However, there can be different ways of contamination; some of them also refer to the plastic injection molding processes and the way the part is obtained. If the mold is cooled with oil, there is always the risk that the oil can escape through the seals and contaminate the amorphous structure of the injected material. Additionally, the oil lubrication of the moving parts of the injection molding machine could be a possible cause of contamination, as it may not be sufficiently cleaned before application of the UV-cured clear lacquer. Handling of parts at the plastics injection molding shop, intermediate storage, and dragging them onto the coating line can contaminate and lead to the occurrence of surface defects.
- A future research direction would be to investigate and fit the proposed method for detecting failure modes in UV-cured clear-coated composite parts obtained by the autoclave curing process.

## Figures and Tables

**Table 1:** Factors and their levels for Taguchi DoE.

Levels	Mold Temp	Mold Pressure	3D Shape	Surface Tension	Primer Thickness	Laser Current	Clear CoatThickness	UV Power
	°C	bar	mm	mN/m	micron	A	micron	%
**Level 1**	65	825	0.1	30	16	60	28	30
**Level 2**	70	830	0.2	32	18	70	30.5	37
**Level 3**	80	835	0.3	35	20	80	32	45

**Table 2:** The tests and the results.

No.	To Do	How Many?	Result	Remarks
1	Surface tension test by Dyne pens	8 frames	The affected area is for 30 mN/m, and the good one is for 35 mN/m	Each frame with 2 × 2 cavities out of 8 cavities
2	Visual test: sink marks, scratches—on injected parts	8 frames	All parts OK, no defects detected	Each frame with 2 × 2 cavities out of 8 cavities
3	Microscopic optical test: pin holes, fish-eyes—on injected parts	8 frames	All parts OK, no defects detected	Each frame with 2 × 2 cavities out of 8 cavities
4	Scan 3D GOM each cavity out of 8 cavities	3 × 8 cavities	Cavities 3, 4, 5, and 6 have been observed with bigger deformations compared with the ones from the cavities 1,2, 7, and 8	One for each cavity was measured in GOM 3D
5	Adhesion test	3 parts	Nothing detected, Level 1	Each frame with two cavities out of 8 cavities
6	Thickness test	4 metallic plates	The thickness in tolerance field, average of 17 microns	
7	Visual test: orange peel, scratches, inclusions, pin holes after the black primer coating	8 frames	1 pc. Not OK—inclusions due to contamination	Not linked with the fish-eye defect
8	Microscopic optical test: fish-eyes—after the black primer coating	8 frames	6 pcs. Not OK—fish-eye present in the rectangle window area	
9	Electron microscope with EDX test: material components	5 pcs.	Results presented in the separate chapter	
10	Visual test: incomplete coating removal—after laser engraving	8 frames	No issues detected	
11	Microscopic optical test: cut edge failures after laser engraving	8 frames	1 pc. Not OK—“fish-eye” defect present near the engraved symbol	
12	Thickness test	4 metallic plates	The thickness in tolerance field, average of 31 microns	
13	Microscopic optical test: fish-eyes, after the UV-cured transparent coat	8 frames	3 pcs. Not OK—fish-eye present in the rectangle window area	

**Table 3:** EDX results for the first area from Figure 9.

	Net Counts				Weight %				Atom %			
	C-K	N-K	O-K	Si-K	C-K	N-K	O-K	Si-K	C-K	N-K	O-K	Si-K
Pt1	1840	335	205	3	41.37	46.86	11.56	0.21	45.80	44.49	9.61	0.10
Pt2	2566	393	177	3	45.79	45.94	8.12	0.15	50.13	43.13	6.67	0.07
Pt3	197	130	73	0	19.61	62.59	17.80	0.00	22.64	61.94	15.42	0.00
Pt4	1589	332	190	0	38.94	49.34	11.72	0.00	43.25	46.99	9.77	0.00

**Table 4:** Response Table for Signal to Noise Ratios/Smaller is better.

Level	Mold Temp	Mold Pressure	3D Shape	Surface Tension	Primer Thickness	Laser Current	Clear Coat Thickness	UV Power
1	−1338	−2398	−4127	−4519	−3181	−1729	−4127	−4519
2	−1729	−3736	−3850	−3458	−3458	−2398	−3181	−2121
3	−7308	−4241	−2398	−2398	−3736	−6248	−3067	−3736
Delta	5970	1843	1729	2121	0555	4519	1060	2398
Rank	1	5	6	4	8	2	7	3

## Data Availability

Not applicable.
